# Presence of the neonatal *Staphylococcus capitis* outbreak clone (NRCS-A) in prosthetic joint infections

**DOI:** 10.1038/s41598-020-79225-x

**Published:** 2020-12-28

**Authors:** Staffan Tevell, Sharmin Baig, Bengt Hellmark, Patricia Martins Simoes, Thierry Wirth, Marine Butin, Åsa Nilsdotter-Augustinsson, Bo Söderquist, Marc Stegger

**Affiliations:** 1 Department of Infectious Diseases, Karlstad Hospital and Centre for Clinical Research and Education, Värmland County Council, SE-65182 Karlstad, Sweden; 2 School of Medical Sciences, Faculty of Medicine and Health, Örebro University, Örebro, Sweden; 3 Department of Bacteria, Parasites and Fungi, Statens Serum Institut, Copenhagen, Denmark; 4 Department of Laboratory Medicine, Faculty of Medicine and Health, Örebro University, Örebro, Sweden; 5 Department of Bacteriology, Institute for Infectious Agents, National Reference Center for Staphylococci, Hospices Civils de Lyon, Lyon, France; 6 Centre International de Référence en Infectiologie, INSERM U1111, CNRS UMR 5308, ENS, University of Lyon, Lyon, France; 7 Institut de Systématique, Evolution, Biodiversité (ISYEB), UMR-CNRS 7205, Muséum National d’Histoire Naturelle, CNRS, EPHE, Sorbonne Université, Paris, France; 8 École Pratique des Hautes Études, PSL Université, Paris, France; 9 Neonatal Intensive Care Unit, Hôpital Femme Mère Enfant, Hospices Civils de Lyon, Lyon, France; 10 Division of Inflammation and Infection, Department of Infectious Diseases, Linköping University, Norrköping, Sweden; 11 Division of Inflammation and Infection, Department of Biomedical and Clinical Sciences, Linköping University, Norrköping, Sweden

**Keywords:** Bacterial genetics, Clinical microbiology, Infectious diseases, Bacterial infection

## Abstract

*Staphylococcus capitis* is a coagulase-negative staphylococcus that has been described primarily as causing bloodstream infections in neonatal intensive care units (NICUs), but has also recently been described in prosthetic joint infections (PJIs). The multidrug-resistant *S. capitis* subsp. *urealyticus* clone NRCS-A, comprising three sublineages, is prevalent in NICUs across the world, but its impact on other patient groups such as those suffering from PJIs or among adults planned for arthroplasty is unknown. Genome sequencing and subsequent analysis were performed on a Swedish collection of PJI isolates (n = 21), nasal commensals from patients planned to undergo arthroplasty (n = 20), NICU blood isolates (n = 9), operating theatre air isolates (n = 4), and reference strains (n = 2), in conjunction with an international strain collection (n = 248). The NRCS-A Outbreak sublineage containing the composite type V SCC*mec*-SCC*cad/ars/cop* element was present in PJIs across three Swedish hospitals. However, it was not found among nasal carrier strains, where the less virulent *S. capitis* subsp. *capitis* was most prevalent. The presence of the NRCS-A Outbreak clone in adult patients with PJIs demonstrates that dissemination occurs beyond NICUs. As this clone has several properties which facilitate invasive infections in patients with medical implants or immunosuppression, such as biofilm forming ability and multidrug resistance including heterogeneous glycopeptide-intermediate susceptibility, further research is needed to understand the reservoirs and distribution of this hospital-associated pathogen.

## Introduction

Prosthetic joint infection (PJI) is a dreaded complication of arthroplasty, resulting in considerable suffering for the patient and increased costs for healthcare providers^1,2^. Since the incidence of PJIs appears to be increasing^1,3^, there is a need for efforts to improve our knowledge in this field regarding prevention, diagnosis, and treatment^4^. Staphylococci are the predominant cause of PJIs. *Staphylococcus aureus* is the most common, followed by *Staphylococcus epidermidis* and *Staphylococcus capitis*^5^, with the latter consisting of two subspecies: subsp. *capitis* and subsp. *urealyticus*. These three staphylococci differ regarding the presence of virulence factors and multidrug resistance (MDR), as methicillin and rifampin resistance are far less prevalent in *S. aureus* and *S. capitis* than in *S. epidermidis*^6–8^. Despite this, recent publications have raised concerns about the outcome after debridement, antibiotics, and implant retention (DAIR) in PJIs caused by *S. aureus*^6,8,9^. There are only few clinical reports on bone and joint infections caused by *S. capitis*^10,11^, and the clinical characteristics of PJIs caused by *S. capitis* have only recently been described. One study showed that 70% of infections were cured following DAIR, and the all-cause 12-month mortality was ≤ 5%^7^. It has been suggested that there are differences in biofilm formation and prevalence of MDR between the two *S. capitis* subspecies^12^.

Most reports on *S. capitis* focus on its ability to cause late-onset sepsis (LOS) in newborns at neonatal intensive care units (NICUs)^13^. In particular, the NRCS-A clone is found worldwide in NICUs^14,15^. Wirth et al*.*^16^ described the differences between Basal *S. capitis*-strains and the NRCS-A clone and explored the evolution and genetic characteristics behind the success of this clone in NICUs. In that recent study, three sublineages of NRCS-A: Proto-Outbreak 1 (POB1), Proto-Outbreak 2 (POB2), and Outbreak were defined, and of these the methicillin-resistant, heterogeneous glycopeptide-intermediate *S. capitis* (hGISC) Outbreak sublineage dominated in NICUs. Its genetic trait characteristics include *tarFIJL*, involved in production of wall teichoic acids (WTA) that are important in biofilm formation, attachment to biomaterials, and protection against cell damage (e.g. glycopeptide resistance)^17,18^; *nsr* (nisin resistance), which has been suggested to be involved in gut colonization preceding LOS among neonates^19^; and a type V SCC*mec*-SCC*cad/ars/cop* element (V-NRCS-A) including a type III-A CRISPR element. Recent years have seen an increasing amount of available *S. capitis* genome data. However, as most of these isolates are either associated with the NICU or insufficiently described, no conclusions can be drawn regarding the presence of the NRCS-A clone either in PJIs or in the community.

The aim of the present study was to investigate the genetic relatedness and diversity among *S. capitis* isolated from PJIs compared to commensal and neonatal isolates in relation to the NRCS-A clone.

## Results

### Genome sequencing and phylogenetic analysis

**Figure 1:**
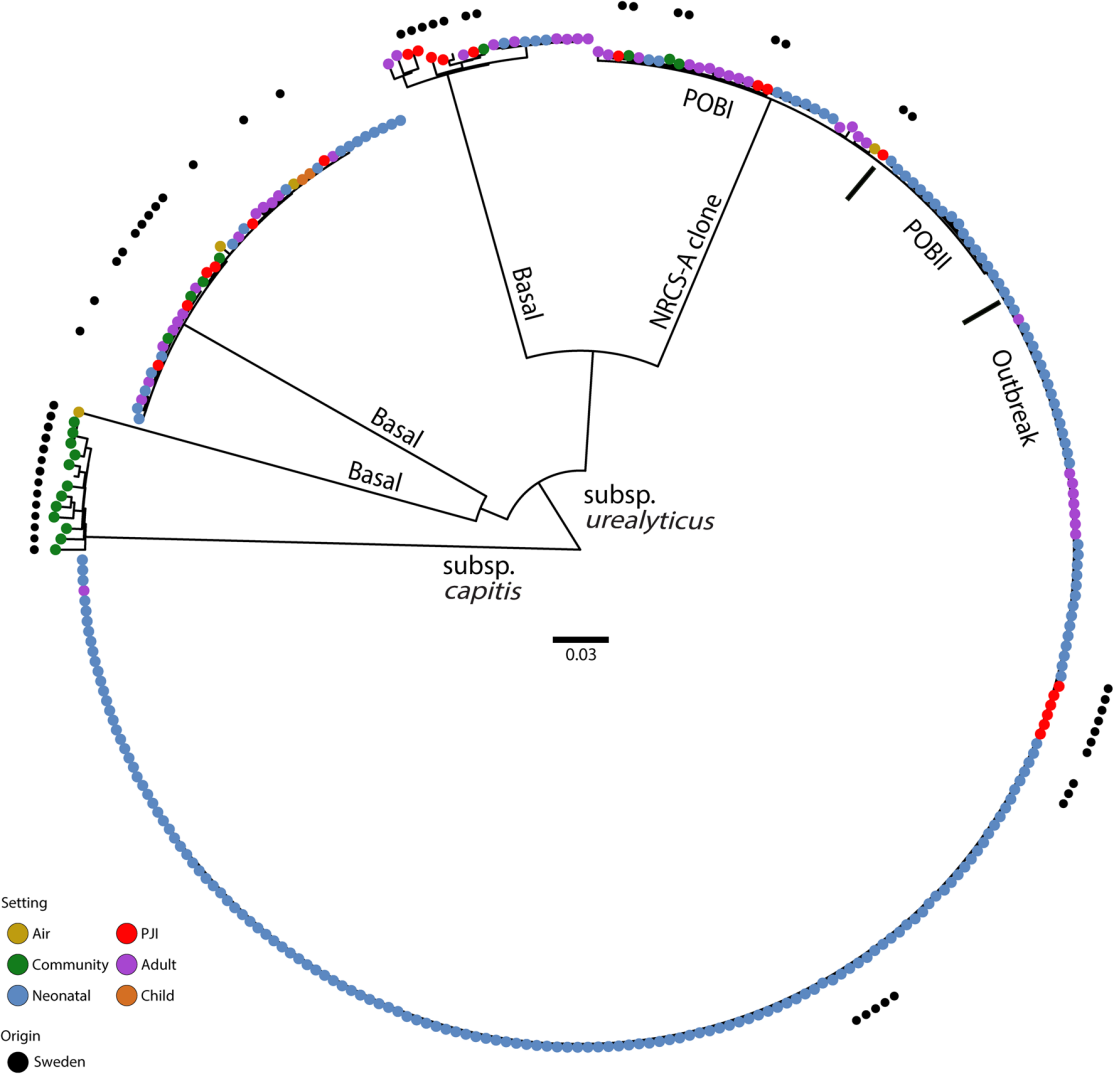
Midpoint-rooted maximum-likelihood phylogeny of 305 *S. capitis* isolates based on 37,970 SNPs after purging of recombination. The Swedish isolates are represented by black dots. The colours in the main circle describe the setting where isolates were retrieved: yellow = air, green = community, blue = neonatal, red = PJI, purple = adult (unspecified location), brown = child (unspecified location). The subspecies differentiation of *S. capitis* is presented, as are the sublineages of *S. capitis* subsp. *urealyticus*. *POBI* proto-outbreak 1. *POBII* proto-outbreak 2. Scale bar indicates substitutions per site.

The obtained Illumina sequencing data from the 56 Swedish isolates were assembled into draft genomes with an average sequencing depth of 172 (all > 42 fold), and with between 45 and 357 contigs (average 141). The genome size varied between 2.3 to 2.6 Mb. Using the raw sequencing data of the Swedish isolates with sequence data from the international collection, 82,969 SNPs were obtained in a core genome of ~ 1.8 Mb (72%) which reduced to 37,970 SNPs after removing recombinant regions. In contrast to the nine Swedish NICU strains, which clustered exclusively with the international NRCS-A Outbreak sublineage, PJI isolates were intermingled throughout all previously defined clades including the Outbreak sublineage (Fig. 1). Also present was a highly distinct clade containing nasal commensals only. These isolates were generally antibiotic susceptible; however, three of them also unexpectedly harboured an *nsr* gene variant.

**Figure 2:**
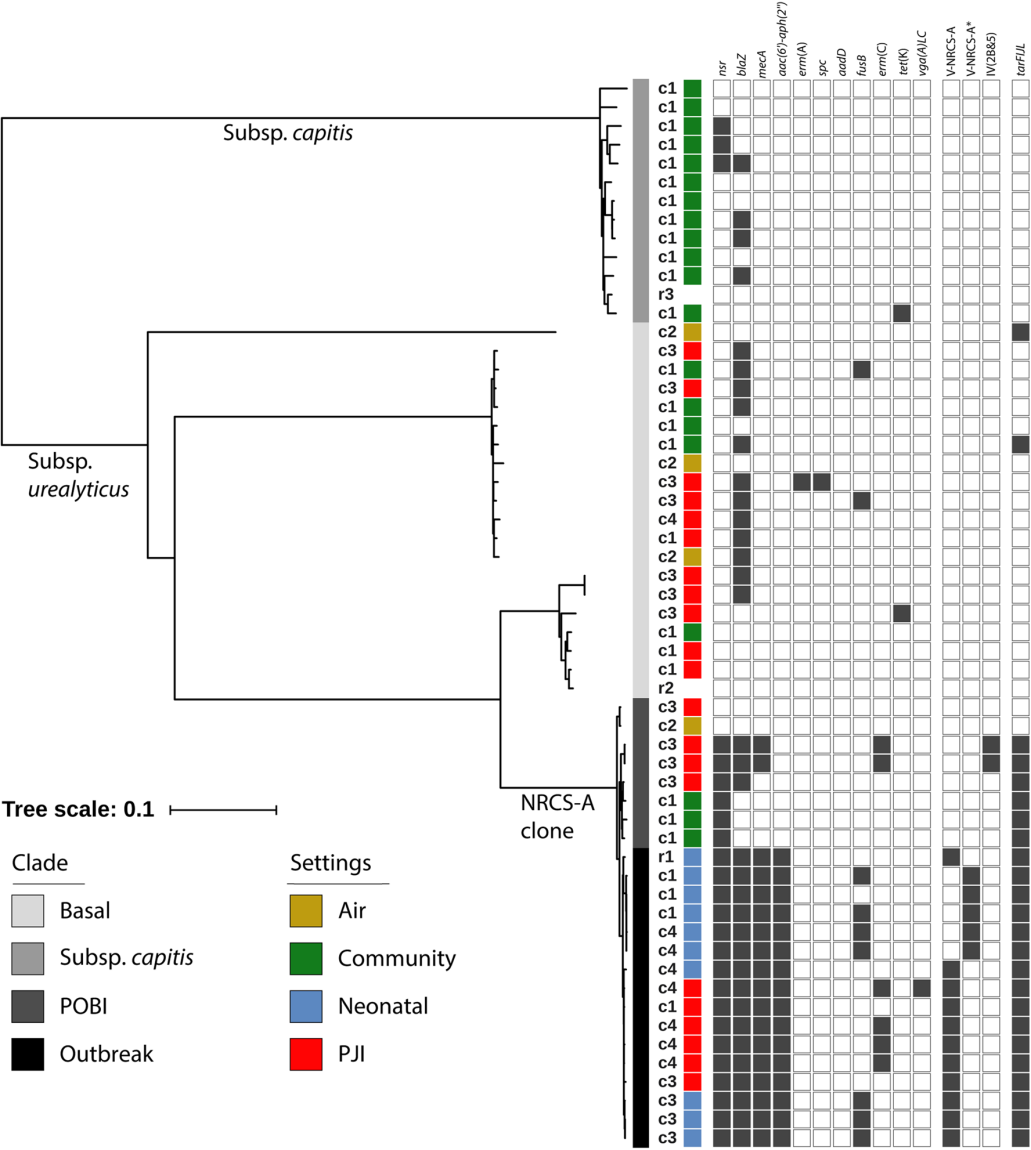
Midpoint-rooted maximum-likelihood phylogeny of 56 *S. capitis* isolates and presence of resistance genes based on a 75% core genome. Different centres are identified as c1–c4 and reference isolates named r1 (NRCS-A prototype strain CR01), r2 (CCUG 55892), and r3 (CCUG 35173). All isolates except r1 were isolated in Sweden. The settings where all Swedish strains were isolated are presented in colour: yellow = air, green = community, blue = neonatal, red = PJI, and white = no data. From c1 was included nasal isolates (n = 20), PJI isolates (n = 4) and NICU isolates (n = 3), from c2 operating theatre air (n = 4), from c3 PJI isolates (n = 12) and NICU isolates (n = 3) and from c4 PJI isolates (n = 5) and NICU isolates (n = 3). The phylogeny highlighted the subsp. *capitis*, Basal, and NRCS-A clade sublineages. Black blocks represent presence of genes mediating antibiotic resistance and SCC*mec* type.

The Swedish-only phylogeny, based on 29,811 purged SNPs obtained from an initial 79,018 SNPs identified in a ~ 1.9 Mb (75%) core genome, revealed the presence of three distinct clades (Fig. 2). The uppermost clade (subsp. *capitis*) was distinctly separated from the other two clades. This clade included the CCUG 35173 *S. capitis* subsp. *capitis* reference strain as well as most of the commensal isolates. The middle clade (Basal lineage) was more diverse and included commensals, PJI isolates, operating theatre air isolates, and the CCUG 55892 *S. capitis* subsp. *urealyticus* reference strain, but no NICU-associated isolates. The lower clade (NRCS-A, containing the POB1, POB2, and Outbreak sublineages) contained all the NICU-associated isolates, 10 isolates from PJIs, and one isolate from operating theatre air. All 18 *mecA*-positive isolates were part of the NRCS-A clade. The composite type V SCC*mec*-SCC*cad/ars/cop* element was found in 11 out of 18 Swedish *mecA* positive isolates, including six from PJIs located in the Outbreak sublineage. Five isolates, all from NICUs in two of the participating centres, had a partial deletion of the element (including the *ccrA1* and *ccrB3* genes) marked as V-NRCS-A* in Fig. 2. In addition, two isolates carried SCC*mec* type IV(2B&5).

The 29 genes previously associated with the NRCS-A clade^16^ were also mostly found in the NRCS-A clone, but two genes found in the SCC*mec* composite element (CR01_v3_0458 and CR01_v3_0459) were also found in two isolates in the subsp. *capitis* clade and two isolates in the Basal clade.

The *nsr* gene was present in all but two POB1 isolates in the NRCS-A clade, together with an additional three isolates in the subsp. *capitis* clade. However, the homology to the previously described NRCS-A *nsr* gene was 99.77% for those three isolates, and further analysis revealed four non-synonymous changes, one resulting in a premature stop codon. All isolates in the NRCS-A clade with the exception of two POB1 isolates, but also two isolates in the Basal clade carried *tarFIJL* genes. Within the NRCS-A clade, seven of the ten PJI isolates were hGISC, and all seven were *tarFIJL*-positive, compared to the Basal clade where one of the 12 PJI isolates was hGISC and none were *tarFIJL*-positive (*p* = 0.002, Fisher’s exact test). Further clinical and phenotypical data on the NRCS-A Outbreak sublineage PJI isolates are presented in Table 1.

**Table 1:** Clinical data for the PJI NRCS-A Outbreak isolates.

Centre	Infection type	Year of surgery	Year of diagnosis	MDR	hGISC	Biofilm CRA/MTP
c3	Chronic, polymicrobial	2001	2010	−	+	+ /−
c4	Chronic, monomicrobial	2009	2010	+	+	+ / +
c4	Chronic, monomicrobial	2011	2011	+	−	+ /−
c4	Early, polymicrobial	2011	2011	+	+	+ /−
c1	Early, polymicrobial	2011	2011	−	+	+ / +
c4	Early, monomicrobial	2012	2012	+	+	+ /−

*MDR* multi-drug resistant isolate, *hGISC* heterogeneous glycopeptide intermediate *S. capitis*, *CRA* Congo red agar, *MTP* microtitre plate assay.

### Bacterial subspecies

In silico analysis of the 16S rRNA gene did not discriminate between the two subspecies. All Swedish isolates in the subsp. *capitis* clade were identified as *S. capitis* subsp. *capitis* based on urease activity and maltose fermentation tests, including reference strain CCUG 35173. Among the isolates in the Basal and NRCS-A clades, 16/43 (37%) isolates (including the reference strain CCUG 55892) were urease-negative/maltose-positive while the remaining 27 (63%) were urease-positive/maltose-positive. These results were consistent on re-analysis, and urease-negative strains were scattered across the phylogeny (see Supplementary Fig. S1 online), indicating unreliable performance of the urease activity test within one subspecies. Thus, the maltose-positive isolates were interpreted as subsp. *urealyticus* regardless of the urease activity.

## Discussion

Here we have demonstrated that the *S. capitis* NRCS-A Outbreak clone is not solely restricted to NICU-associated sepsis, but also occurs in adult PJIs in all three investigated Swedish regions. The NRCS-A clone has been shown capable of causing outbreaks in NICUs worldwide, but it is not currently known whether these cases in orthopaedic implant surgery represent spillover from NICUs, or a more widespread in-hospital dissemination than previously reported. However, the temporal distribution indicates that an outbreak situation of the kind seen in NICUs is presently unlikely.

The major specific genetic features that have been described for this clone in NICUs are a type V SCC*mec*-SCC*cad/ars/cop,* alteration of WTAs through *tarFIJL,* and nisin resistance^16^*.* When revisiting clinical and phenotypic data^7^, MDR (including methicillin resistance and hGISC) and biofilm production were common among the NRCS-A PJI Outbreak isolates. Virulence factors, such as biofilm formation, immune evasion, and antibiotic resistance, have an impact on pathogenesis and treatment strategies in PJIs, and the NRCS-A Outbreak clone contains all characteristics required for a successful PJI pathogen.

The *nsr* gene was present in all isolates in the NRCS-A Outbreak clade, as well as the composite type V SCC*mec*-SCC*cad/ars/cop* element. However, the presence of SCC*mec* type IV in two Proto-Outbreak isolates showed at least two independent acquisitions of SCC*mec*. In PJIs, methicillin resistance mediates a lack of susceptibility to first-line prophylaxis (i.e. cefazolin or cloxacillin), and hGISC mediates reduced susceptibility to first-line treatment in methicillin-resistant coagulase-negative staphylococci. Several functions have been proposed for WTA in *S. aureus*, including interaction with biomaterials and receptors (e.g. mediating adherence to epithelial and endothelial cells, attachment to biomaterials, and biofilm formation) and protection against cell damage (e.g. resistance to vancomycin and lysozyme)^17,18^. Interestingly, the presence of *tarFIJL* was significantly associated with hGISC expression when comparing the PJI isolates in the Basal clade to those in the NRCS-A clade. Formation of biofilm is crucial in adherence to surfaces, both for persistence in the environment, which is implied to be of importance in the NICU^15^, and in the pathogenesis of PJIs. Nisin resistance, however, may possibly be beneficial for survival in the gut among neonates^19^, but there is no obvious connection between nisin resistance and pathogenesis of PJIs. Thus, nisin resistance may simply be a fortuitous passenger in a virulent nosocomial strain residing on surfaces in the NICU, with potential for nosocomial spread. Further environmental sampling from hospital surfaces outside the NICU is required to determine potential reservoirs for persistence of nosocomial *S. capitis* strains, which could improve preventive measures against the devastating infections PJIs constitute.

Nasal colonization with *S. aureus* has been described as a risk factor for surgical site infections such as PJIs^20^, and nasal and PJI strains are phylogenetically similar^8^. However, a discrepancy between the antibiotic susceptibility and sequence types among commensal and PJI isolates of *S. epidermidis* indicates that the nares are colonized with different strains from those causing PJIs^21,22^. The majority of the nasal isolates in the present study belonged to antibiotic susceptible subsp. *capitis,* and lack of several of the virulence genes found in the NRCS-A clade, thus indicating that strains with lower virulence are prevalent in the community. Still, although commensal isolates were diverse and consisted of both subspecies, the NRCS-A Outbreak sublineage was not found in nares. Thus, based on this collection of isolates, nasal colonization does not seem to be a major source for nosocomial infections by the NRCS-A clone.

Subtyping for differentiation between the two subspecies is generally not performed in clinical practice. When comparing the WGS-based clustering with phenotypic assays, urease activity proved unreliable; all the subsp. *capitis* isolates and 37% of the subsp. *urealyticus* were negative, which is in contrast to data from Bannerman et al*.*^23^, where > 90% of subsp. *urealyticus* were positive. However, the maltose fermentation test alone could discriminate between the two subspecies in all tested isolates. While subsp. *urealyticus* apparently can truly act both as commensal and as pathogen, the pathogenic potential of subsp. *capitis* is uncertain. If future research confirms the presence of clades with different disease-causing potential in PJIs, determining which clade a clinical isolate belongs to may help discriminate commensals from pathogens, as subsp. *capitis* seems to have lower pathogenic potential than subsp. *urealyticus*.

Limitations of the present study include the small cohort size; however, the identification of NRCS-A strains in PJIs in all three investigated centres despite the limited cohort size is alarming. Additionally, no data were available regarding prior healthcare contact of nasal carriers of subsp. *urealyticus*, particularly the POB1 sublineage. Future research focusing on nasal colonization in subjects with diverse and known backgrounds may shed further light on nasal carriage of different subspecies/clones of S. *capitis*.

In conclusion, the NRCS-A clone is not exclusive to NICUs, but is also able to cause serious infections, such as PJIs, in adults. Further research is needed to fully understand reservoirs and distribution among patients at risk for nosocomial opportunistic infections, including immunocompromised patients and those with medical implants.

## Methods

### Bacterial isolates

Whole-genome sequencing (WGS) was performed on 54 *S. capitis* isolates from three adjacent Swedish regions: Värmland, Örebro, and Östergötland, encompassing approximately one million inhabitants. The clinical and demographic characteristics of the PJI isolates (n = 21) have been reported previously^7^. Briefly, these were isolated from tissue biopsies taken during reoperations or revisions of infected prosthetic joints between 2005 and 2014. Nasal isolates (n = 20) were obtained prior to hospital admission, at first outpatient clinic visit during 2017–2018 from patients planned for elective hip or knee replacement surgery. No clinical data were available for these patients. Three isolates from epidemiologically unrelated cases of *S. capitis*-bacteraemia in the NICU were selected from each of the participating regions during 2014–2016 (n = 9). As the primary aim was to determine whether the MDR NRCS-A clone was present in the NICUs, MDR strains were randomly selected if more than three isolates were available. No clinical data were collected for these patients, so it was not known whether LOS was present. Isolates from operating theatre air (n = 4), collected in 2011–2012 in the adjacent region of Västmanland^24^ were also included. All isolates were determined to species level using MALDI-TOF MS (Microflex LT and Biotyper 3.1; Bruker Daltonics, Bremen, Germany). Also included were published genomic data from an international collection of 250 isolates^16^ available at the Sequence Read Archive (https://www.ncbi.nlm.nih.gov/sra) BioProject number PRJNA493527; two of these were excluded after quality assessment. Additionally, subspecies reference isolates CCUG 35173 (*S. capitis* subsp. *capitis*) and CCUG 55892 (*S. capitis* subsp. *urealyticus*) obtained from the University of Gothenburg Culture Collection (http://www.ccug.se) were genome sequenced and included.

### Genome sequencing and phylogenetic analysis

Genomic DNA was purified using the QIAGEN Blood and Tissue Kit (Qiagen). A sequencing library was produced using a Nextera XT kit (Illumina) according to the manufacturer’s instructions, followed by paired-end sequencing using 300- or 500-cycle kits on a MiSeq or NextSeq instrument (Illumina), respectively. The resulting datasets are available from the SRA under BioProject number PRJEB35698. Genome sequences were de novo assembled using SPAdes v3.11.1^25^ with default parameters.

To examine their ability to discriminate between *S. capitis* subsp. *capitis* and subsp. *urealyticus,* subspecies relations were investigated using in silico analysis of 16S rRNA genes, as well as urease activity and maltose fermentation tests^23^ using the ID32 STAPH system (bioMérieux, Marcy l’Etoile, France) according to the manufacturer’s instructions*.*

Single nucleotide polymorphisms (SNPs) were identified using NASP v1.0.0^26^. BWA-MEM was used to align Illumina reads from individual isolates against the chromosome of *S. capitis* isolate CR01 (GenBank accession number LN866849). Positions with ≤ 10-fold sequencing depth and/or < 90% unambiguous variant calls were removed using GATK after positions within duplicated or repetitive regions of the reference genome were masked using NUCmer. Two SNP-based analyses were performed, one for the Swedish-only collection (n = 56) and one including the additional 248 isolates. For both analyses, recombinant regions were removed using Gubbins v.2.3.4^27^. Phylogenetic trees of all isolates were constructed using the maximum-likelihood approach in PhyML v3.3 with the GTR substitution model and 100 bootstrap replicates^28^. The phylogenies were visualized using iTOL (https://itol.embl.de).

Resistance genes were detected from the assembled draft genes with ABRicate (https://github.com/tseemann/abricate) using the ResFinder (cge.cbs.dtu.dk/services/ResFinder/) database. In addition, the presence of 29 previously identified genes associated with the Outbreak/Proto-Outbreak strains^16^, including the *nsr* gene encoding nisin resistance and a cell wall teichoic-acid associated gene cluster (*tarFIJL*), were examined using a BLASTN search against the assembled genomes.

For the *mecA*-positive Swedish isolates, the SCC*mec* elements were identified using SCC*mec*Finder^29^ with minimum 40% coverage and 80% identity. In addition, reads were mapped towards the composite SCC*mec* element described by Martins Simoes et al. (GenBank accession number KF049201)^30^.

### Ethics approval

Access to clinical data (reference: 2014/418) and the collection of commensal isolates (reference: 2012/092) were approved by the Regional Ethical Review Board of Uppsala.

### Consent for publication

Not applicable.

## Supplementary Information

Supplementary Figure S1.

## Data Availability

The datasets generated and analysed during the current study are available from the SRA under BioProject number PRJEB35698.

## Abbreviations

DAIR: Debridement, antibiotics, and implant retention
hGISC: Heterogeneous glycopeptide-intermediate *S. capitis*
LOS: Late-onset sepsis (in neonates)
MDR: Multidrug resistance
NICUs: Neonatal intensive care units
PJIs: Prosthetic joint infections
POB1: Proto-outbreak 1
POB2: Proto-outbreak 2
WGS: Whole-genome sequencing
WTA: Wall teichoic acids
