# Supplementary material for: Presence of the neonatal Staphylococcus capitis outbreak clone (NRCS-A) in prosthetic joint infections
Source: Sci Rep. 2020 Dec 28;10:22389. doi: 10.1038/s41598-020-79225-x (PMC7769963; doi:10.1038/s41598-020-79225-x)

**Supplementary material to:**

"Presence of the neonatal *Staphylococcus capitis* Outbreak clone (NRCS-A) in prosthetic joint infections"

S. Tevell, S. Baig, B. Hellmark, P. Martins Simoes, T. Wirth, M. Butin, Å. Nilsdotter-Augustinsson, B. Söderquist, M. Stegger

**Supplementary Figure S1: Midpoint rooted maximum-likelihood phylogeny of *S. capitis* isolates and results of urease activity and maltose fermentation tests**

Midpoint rooted maximum-likelihood phylogeny of 56 Swedish *S. capitis* isolates and the NRCS-A prototype strain CR01, including results of urease activity and maltose fermentation tests. White blocks represent negative test and black blocks represent positive test. Setting was not available for reference isolates r2 and r3, and urease activity and maltose fermentation tests was not performed on reference strain CR01, as only sequence data was available.

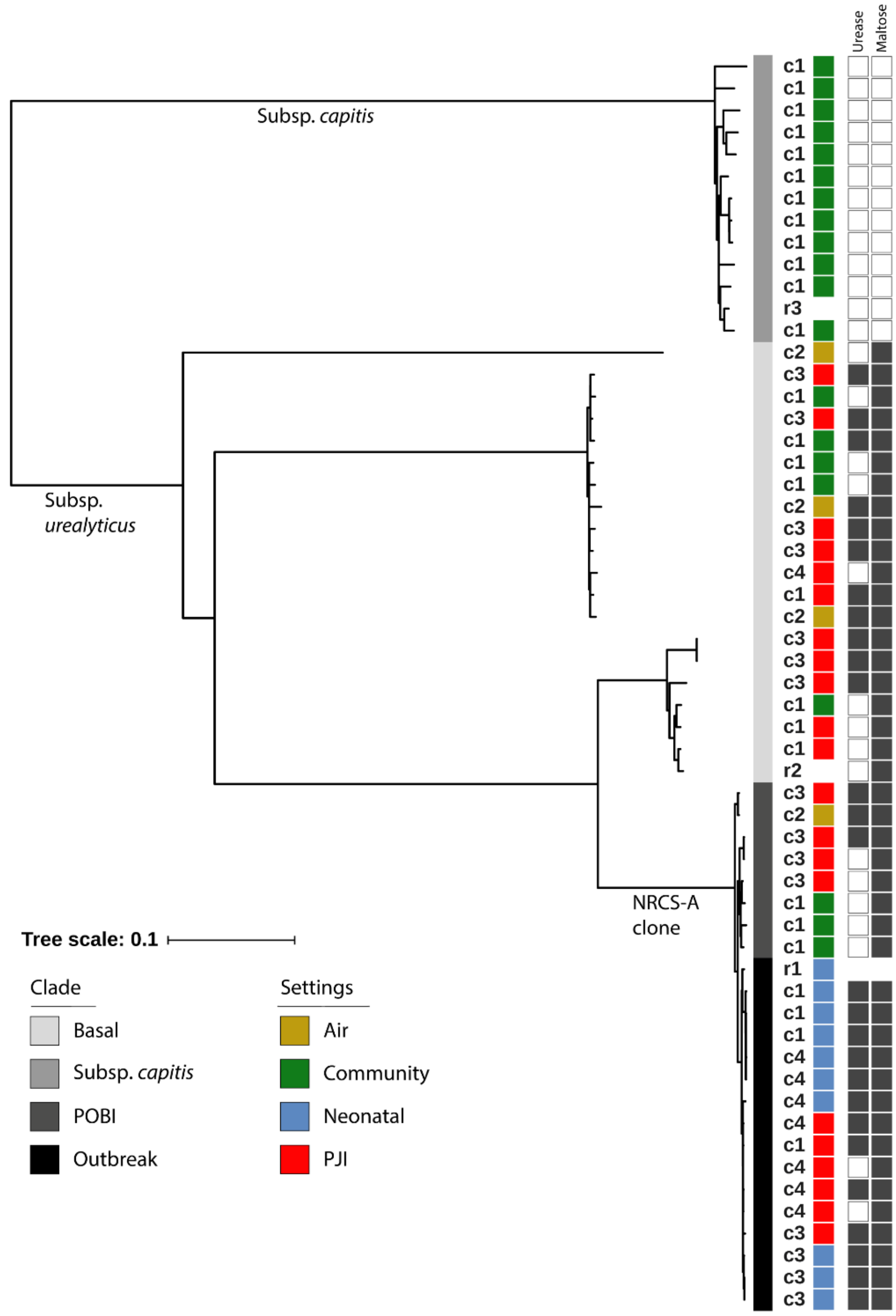

Supplement: Supplementary file 1 — Supplementary Figure S1. [file 41598_2020_79225_MOESM1_ESM.pdf]
